# Obesity, abdominal obesity and subsequent risk of kidney cancer: a cohort study of 23.3 million East Asians

**DOI:** 10.1038/s41416-019-0500-z

**Published:** 2019-06-24

**Authors:** Ga Eun Nam, Kyung Hwan Cho, Kyungdo Han, Chul Min Kim, Byoungduck Han, Sung Jung Cho, Seung Jin Jung, Yeongkeun Kwon, Yang Hyun Kim, Do Hoon Kim, Seon Mee Kim, Youn Seon Choi, Yong Kyun Roh, Yong Gyu Park

**Affiliations:** 1 Department of Family Medicine, Korea University College of Medicine, Seoul, Republic of Korea; 2 Department of Biostatistics, College of Medicine, The Catholic University of Korea, Seoul, Republic of Korea; 3 Department of Family Medicine, College of Medicine, The Catholic University of Korea Seoul, Seoul, Republic of Korea; 4 Department of Family Medicine, Sahmyook Medical Center, Seoul, Republic of Korea; 5 Department of Family Medicine, Kangnam Sacred Heart Hospital, Hallym University College of Medicine, Seoul, Republic of Korea

**Keywords:** Cancer epidemiology, Cancer prevention

## Abstract

**Background:**

Limited evidence exists regarding associations between obesity and kidney cancer among Asians. We examined the associations between obesity measures and risk of kidney cancer.

**Methods:**

We included 23,313,046 adults who underwent health examinations provided by the Korean National Health Insurance Service 2009–2012 and performed multivariable Cox proportional hazards regression analyses.

**Results:**

During 5.4 years of follow-up, 18,036 cases of kidney cancer were recorded, and cumulative incidence was 0.12%. General and abdominal obesity were associated with 1.32-fold increased risk of kidney cancer compared with groups without either obesity status. Underweight individuals showed decreased adjusted hazard ratio (HR) for kidney cancer (0.76, 95% confidence interval: 0.68–0.85) compared to those with normal body mass index (BMI), while the HRs increased among individuals with BMI 23–24.9 kg/m^2^ (1.23, 1.18–1.28), 25–29.9 kg/m^2^ (1.41, 1.36–1.46) and ≥30 kg/m^2^ (1.77, 1.65–1.90) (*P* for trend < 0.001). HRs of kidney cancer increased with increasing waist circumference (WC) (*P* for trend < 0.001). Compared to non-obese condition, the coexistence of general and abdominal obesity increased the HR (1.45, 1.40–1.50).

**Conclusions:**

This study demonstrated positive associations of BMI and WC with kidney cancer risk. General and abdominal obesity may be risk factors of kidney cancer.

## Background

Kidney cancer represents ~2% of all cancers worldwide and is the 9th and 14th most common type of cancer in men and women, respectively. Globally, the incidence and mortality rate of kidney cancer have been reported to have increased by 2–3% per decade over the past several decades.^1^ To date, kidney cancer has been reported to be less prevalent in the East compared with that in the West. However, the incidence is increasing rapidly in Asian populations in line with the prevalence of obesity. In Korea, kidney cancer has been on the rise with an annual percentage change of 6% and has become the second most frequent urological cancer since 2008.^2,3^ Previous studies have reported that risk factors, such as smoking, hypertension, obesity and African American race, have been associated with an increased incidence of kidney cancer.^4^

Although recent studies have shown associations between obesity and the risk of kidney cancer, consistently reported positive associations between obesity and renal cell carcinoma (RCC) risk have largely come from studies in the Western population.^5–9^ Evidence on the associations between obesity and kidney cancer in Asian populations is limited. Moreover, most previous studies evaluating the association between obesity and incident kidney cancer have primarily focused on the body mass index (BMI) criteria, while few studies have included waist circumference (WC) in the analysis.^10^ However, BMI does not reflect body composition accurately and therefore, a substantial individual variation in total and visceral adiposity is possible within each category of BMI.^11^ WC is a surrogate parameter for visceral obesity and is less influenced by variation in lean mass.

Thus, this study aimed to evaluate the association between obesity and abdominal obesity (assessed by BMI and WC, respectively), and the risk of subsequent kidney cancer using data from the entire South Korean population.

## Methods

### Study population

This study was based on the database provided by the South Korean National Health Insurance Service (NHIS), which is a single mandatory health insurance system covering nearly the entire South Korean population. The South Korean NHIS provides biannual health examination for all insured Koreans. Hence, it retains a health information of about 50 million Koreans, including data on demographics, health examinations, medical treatment and disease diagnoses according to the International Classification of Disease-10th Revision-Clinical Modification (ICD-10-CM) codes. Since 2006, the South Korean government has implemented a registration program for co-payment reduction for rare, intractable diseases such as cancers. Patients registered in this program are eligible for co-payment reduction after physician’s diagnosis based on the national health insurance diagnostic criteria. The South Korean NHIS has been releasing dynamic, nationally representative, retrospective cohort datasets encompassing the whole South Korean population; the database is open to all researchers whose study protocols are approved by the official review committee.

Among 24,062,918 individuals aged ≥20 years who had undergone health examinations provided by the South Korean NHIS between 1 January 2009 and 31 December 2012, we excluded individuals with missing data (*n* = 739,648), and those who had been diagnosed with kidney cancer between 1 July 2005 and enrolment (*n* = 10,224). Finally, 23,313,046 individuals were enrolled and followed-up till 31 December 2015. The mean follow-up duration was 5.4 ± 1.1 years. The study protocol was approved by the Institutional Review Board of the Catholic University of Korea, St. Mary’s Hospital (IRB number: KC16EISI0939).

### Definition of kidney cancer

The study endpoint was kidney cancer occurrence from the index date until the end of 2015. Incident kidney cancer was diagnosed based on the ICD-10-CM code (C64) and registration code (V193).

### BMI and WC categories

Participants’ height, weight and WC were measured, and BMI was calculated by dividing weight (kg) with the square of height (m). General obesity was defined as BMI ≥ 25 kg/m^2^ based on the World Health Organization recommendations for Asian populations.^12^ In addition, participants were categorised into five BMI groups: <18.5 kg/m^2^ (underweight), 18.5–22.9 kg/m^2^ (normal), 23.0–24.9 kg/m^2^ (overweight), 25.0–29.9 kg/m^2^ (class I obese) and ≥30.0 kg/m^2^ (class II obese). Abdominal obesity was defined as WC ≥ 90 cm for men and ≥85 cm for women according to the Asian-specific WC cut-off for abdominal obesity.^13^ Participants were classified into six groups with 5 cm-interval of WC: < 80.0 cm, 80.0–84.9 cm, 85.0–89.9 cm, 90.0–94.9 cm, 95.0–99.9 cm, ≥ 100.0 cm in men and < 75.0 cm, 75.0–79.9 cm, 80.0–84.9 cm, 85.0–89.9 cm, 90.0–94.9 cm and ≥95.0 cm in women.

### Covariates

We assessed participants’ demographic and lifestyle-related information using standardised self-reporting questionnaires. Income level was dichotomised at the lowest quartile. Individuals who smoked ≥100 cigarettes during their lifetime and were currently smoking were defined as current smokers. Alcohol consumption was categorised by questionnaire regarding the frequency of alcohol consumption (how many times do you drink alcohol?) and average amount of alcohol consumed per drinking (how much do you usually drink?). Heavy alcohol drinkers were defined as individuals who consumed ≥30 g of alcohol per day.^14^ Physical activity was categorised on the basis of the following questionnaire: how many days did you do strenuous physical activities such as running, aerobics, fast bicycling, or mountain climbing ≥20 min during the last 7 days? Regular physical activity was defined as ≥1 time/week of strenuous exercise for at least 20 min. Blood pressure (BP) and serum levels of glucose, lipid profile and creatinine were measured after participants’ overnight fasting. Baseline comorbidities were identified based on the combination of past medical history, and clinical and pharmacy codes of ICD-10-CM. We defined hypertension as BP ≥ 140/90 mmHg, or at least one claim/year for an antihypertensive medication prescription under ICD-10-CM codes of I10-I13, I15. Diabetes mellitus (DM) was defined as fasting glucose ≥ 126 mg/dL, or at least one claim/year for an antidiabetic medication prescription under ICD-10-CM codes of E11-E14. Dyslipidaemia was defined by a serum total cholesterol level ≥ 240 mg/dL, or at least one claim/year for a lipid-lowering medication under ICD-10-CM code of E78. The estimated glomerular filtration rate (eGFR) was calculated using the equation from the Modification of Diet in Renal Disease study: eGFR = 175 × serum creatinine^−1.154^ × age^−0.203^ × 0.742 (for women). Chronic kidney disease (CKD) was defined as eGFR < 60 mL/min/1.73 m^2^.^15,16^

### Statistical analysis

SAS software (version 9.4; SAS Institute, Cary, NC, USA) were used for statistical analysis. Baseline characteristics of study participants according to the BMI categories were compared using analysis of variance (ANOVA) for continuous variables or chi-square test for categorical variables. Incidence rates of kidney cancer were calculated by dividing the number of events by 1000 person-years. We performed multivariable Cox proportional hazards regression analyses to evaluate the association of BMI and WC with incident kidney cancer, and calculated hazard ratios (HRs) and 95% confidence interval (CIs). Model 1 was adjusted for age and sex. Model 2 was additionally adjusted for smoking status, alcohol consumption, physical activity, income, eGFR, DM and hypertension with variables from model 1. In model 3, we further adjusted mutually for BMI or WC in addition to variables from model 2. We evaluated the risk of incident kidney cancer according to the coexistence of general obesity and abdominal obesity. We also conducted clinically relevant subgroup analyses and calculated P values for interactions between obesity and subgroups in the development of kidney cancer using Cox regression analysis.

## Results

### Baseline characteristics of study participants by categories of BMI

Table 1 shows the general clinical characteristics of study participants (*n* = 23,313,046) at baseline according to BMI categories. The proportion of men and mean age were the highest in the class I obesity group (BMI: 25–29.9 kg/m^2^). Mean values of cardiometabolic parameters such as systolic and diastolic BP, total cholesterol, triglycerides, low-density lipoprotein cholesterol and fasting plasma glucose increased with an increase in BMI. The prevalence of comorbidities such as hypertension, DM, dyslipidaemia and CKD was higher in the higher BMI groups. The proportions of heavy alcohol drinkers and current smokers were also significantly higher among obese individuals.

**Table 1:** Baseline characteristics of the study population according to BMI categories

	BMI (kg/m^2^)				
	<18.5	18.5–23.0	23.0–24.9	25.0–29.9	≥30.0
*N*	945,859	9,269,581	5,637,175	6,596,150	864,281
*Sex*^a^					
Males	282,053 (29.8)	3,987,644 (43.0)	3,159,100 (56.0)	3,949,283 (59.9)	450,813 (52.2)
Females	663,806 (70.2)	5,281,937 (57.0)	2,478,075 (44.0)	2,646,867 (40.1)	413,468 (47.8)
Age (years)^b^	41.3 ± 17.2	45.8 ± 14.7	49.5 ± 13.7	49.9 ± 13.5	46.9 ± 14
Height (cm)^b^	162.3 ± 8.1	162.9 ± 8.8	163.6 ± 9.3	164 ± 9.7	163.6 ± 10.3
Weight (kg)^b^	46.4 ± 5.2	56.3 ± 7.0	64.4 ± 7.5	72.3 ± 9.1	86.0 ± 11.8
BMI (kg/m^2^)^b^	17.6 ± 0.8	21.1 ± 1.2	24.0 ± 0.6	26.8 ± 1.3	32.0 ± 2.0
WC (cm)^b^	66.1 ± 5.7	74.1 ± 6.3	81.2 ± 5.7	87.3 ± 6.2	96.9 ± 7.6
Systolic BP (mmHg)^b^	113.3 ± 14.2	118.3 ± 14.6	123.3 ± 14.5	126.9 ± 14.6	131.3 ± 15.1
Diastolic BP (mmHg)^b^	71.0 ± 9.4	73.7 ± 9.7	76.6 ± 9.7	78.9 ± 9.9	81.9 ± 10.3
Total cholesterol (mg/dL)^b^	178.0 ± 32.4	188.7 ± 35.2	197.5 ± 36.8	201.8 ± 37.7	204.8 ± 38.7
Triglycerides (mg/dL)^c^	72 (54–98)	88 (63–128)	114 (80–167)	136 (94–198)	151 (106–219)
HDL-C (mg/dL)^b^	63.1 ± 17.9	58.7 ± 19	54.4 ± 19.6	52.1 ± 20.0	50.8 ± 19.5
LDL-C (mg/dL)^b^	99.4 ± 42.7	109.8 ± 43.5	116.9 ± 43.1	118.8 ± 43.4	119.8 ± 43.8
Fasting glucose (mg/dL)^b^	90.9 ± 19.2	94.2 ± 20.8	98.4 ± 23.5	101.5 ± 25.5	105.6 ± 29.5
Creatinine (mg/dL)^c^	0.8 (0.7–0.9)	0.9 (0.7–1.0)	0.9 (0.8–1.0)	0.9 (0.8–1.1)	0.9 (0.8–1.1)
eGFR (mL/min/1.73 m^2^)^b^	94.7 ± 40.7	90.9 ± 39.1	87.9 ± 39.7	87.0 ± 40.0	88.5 ± 40.7
Hypertension^a^	93,924 (9.9)	1,544,189 (16.7)	1,560,872 (27.7)	2,509,365 (38.0)	437,820 (50.7)
Diabetes mellitus^a^	35,240 (3.7)	551,472 (6.0)	547,677 (9.7)	875,261 (13.3)	160,996 (18.6)
Dyslipidemia^a^	57,793 (6.1)	1,186,402 (12.8)	1,188,111 (21.1)	1,786,142 (27.1)	278,827 (32.3)
Chronic kidney disease^a^	44,462 (4.7)	477,342 (5.2)	354,012 (6.3)	464,494 (7.1)	61,885 (7.2)
Current smoker^a^	199,772 (21.1)	2,140,672 (23.1)	1,407,372 (25.0)	1,743,340 (26.4)	239,614 (27.7)
Heavy alcohol drinker^a^	34,306 (3.6)	481,433 (5.2)	390,981 (6.9)	560,407 (8.5)	76,838 (8.9)
Regular exerciser^a^	369,554 (39.1)	4,456,487 (48.1)	2,910,901 (51.6)	3,393,477 (51.5)	424,359 (49.1)
Income (the lowest quartile)^a^	288,290 (30.5)	2,592,628 (28.1)	1,441,347 (25.6)	1,659,489 (25.2)	246,089 (28.5)

*BMI* body mass index, *WC* waist circumference, *BP* blood pressure, *HDL-C* high-density lipoprotein cholesterol, *LDL-C* low-density lipoprotein cholesterol, *eGFR* estimated glomerular filtration rate

^a^Values are presented as number (percentage)

^b^Values are presented as mean ± standard deviation

^c^Values are presented as median (interquartile range) using the Wilcoxon rank-sum test

### Risk of kidney cancer according to BMI and WC categories

**Fig. 1:**
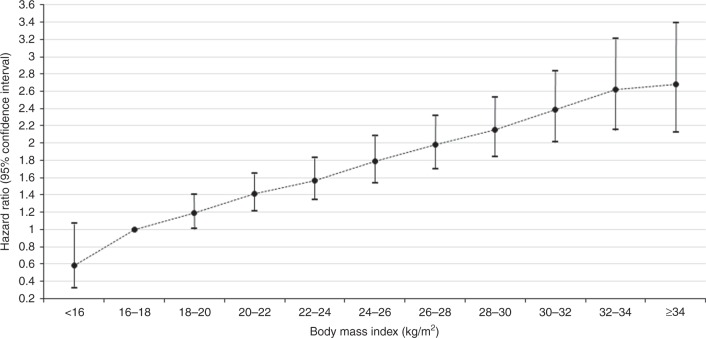
BMI and HR (95% CI) of kidney cancer (*P* < 0.001)

During the mean follow-up of 5.4 years, 18,036 cases of kidney cancer were recorded. Table 2 presents the HRs (95% CIs) of kidney cancer according to BMI and WC categories. Increases in every 1 kg/m^2^ of BMI and 5 cm of WC were significantly associated with 6 and 12.5% increased HR of kidney cancer, respectively. General obesity (BMI ≥ 25 kg/m^2^) and abdominal obesity (WC ≥ 90 cm in men and ≥85 cm in women) were associated with increased risk of kidney cancer compared to individuals without either obesity status after adjusting for confounding variables (HR 1.32, 95% CI: 1.28–1.36 for general obesity; HR 1.32: 95% CI: 1.28–1.37 for abdominal obesity). After adjusting for all confounding variables (model 2), underweight individuals (<18.5 kg/m^2^ of BMI) showed a decreased HR for kidney cancer (0.76, 95% CI: 0.68–0.85) compared to those with a normal BMI (18.5–22.9 kg/m^2^); the HRs increased significantly in the 23.0–24.9 kg/m^2^ (1.23, 95% CI: 1.18–1.28), 25.0–29.9 kg/m^2^ (1.41, 1.36–1.46) and the ≥ 30 kg/m^2^ (1.77, 1.65–1.90) BMI groups. The HRs increased significantly in the higher BMI group (*P* for trend < 0.001). Similar trends were observed for WC categories. HRs of incident kidney cancer increased significantly with increasing WC (*P* for trend < 0.001) and WC ≥ 90 cm (in men) and ≥ 85 cm (in women) was associated with higher risk of kidney cancer. These associations persisted even after further mutual adjustment for WC or BMI (model 3) and in stratified analyses according to sex (Supplemental Table 1 and Supplemental Table 2). In addition, Fig. 1 shows significant positive linear associations between BMI and HRs of incident kidney cancer (*P* < 0.001).

**Table 2:** HR (95% CI) of incident kidney cancer according to BMI and WC categories

	*N*	Event	Person-years	Incidence rate^a^	HR (95% CI)		
					Model 1^b^	Model 2^c^	Model 3^d^
BMI (per 1 kg/m^2^)					1.078 (1.073–1.083)	1.06 (1.055–1.065)	1.037 (1.029–1.044)
BMI (kg/m^2^*)*							
<25.0	15,852,615	10,164	85,236,420	0.12	1 (ref.)	1 (ref.)	1 (ref.)
≥25.0	7,460,431	7872	40,285,267	0.20	1.46 (1.42–1.50)	1.32 (1.28–1.36)	1.11 (1.06–1.15)
*P*					<0.001	<0.001	<0.001
<18.5	945,859	308	4,930,420	0.06	0.72 (0.64–0.81)	0.76 (0.68–0.85)	0.82 (0.73–0.92)
18.5–22.9	9,269,581	5037	49,696,692	0.10	1 (ref.)	1 (ref.)	1 (ref.)
23.0–24.9	5,637,175	4819	30,609,308	0.16	1.29 (1.24–1.34)	1.23 (1.18–1.28)	1.11 (1.07–1.16)
25.0–29.9	6,596,150	6910	35,715,770	0.19	1.56 (1.50–1.61)	1.41 (1.36–1.46)	1.19 (1.13–1.25)
≥ 30.0	864,281	962	4,569,497	0.21	2.13 (1.98–2.28)	1.77 (1.65–1.90)	1.36 (1.24–1.49)
*P*					< 0.001	< 0.001	< 0.001
P for trend					< 0.001	< 0.001	< 0.001
WC (per 5 cm)					1.162 (1.152–1.172)	1.125 (1.115–1.135)	1.091 (1.076–1.106)
WC (cm)							
M < 90.0, F < 85.0	18,611,187	12,114	100,250,905	0.12	1 (ref.)	1 (ref.)	1 (ref.)
M ≥ 90.0, F ≥ 85.0	4,701,859	5922	25,270,782	0.23	1.47 (1.42–1.51)	1.32 (1.28–1.37)	1.12 (1.08–1.17)
*P*					<0.001	<0.001	<0.001
M < 80.0, F < 75.0	8,690,729	3530	46,456,962	0.08	0.65 (0.62–0.68)	0.71 (0.67–0.74)	0.79 (0.75–0.84)
M 80.0–84.9, F 75.0–79.9	5,372,085	4126	29,124,141	0.14	0.87 (0.84–0.91)	0.90 (0.87–0.94)	0.95 (0.91–0.99)
M 85.0–89.9, F 80.0–84.9	4,548,373	4458	24,669,803	0.18	1 (ref.)	1 (ref.)	1 (ref.)
M 90.0–94.9, F 85.0–89.9	2,720,487	3201	14,701,543	0.22	1.12 (1.07–1.18)	1.09 (1.04–1.14)	1.05 (1.00–1.10)
M 95.0–99.9, F 90.0–94.9	1,256,958	1700	6,745,304	0.25	1.30 (1.23–1.38)	1.22 (1.15–1.29)	1.15 (1.08–1.22)
M ≥ 100.0, F ≥ 95.0	724,414	1021	3,823,935	0.27	1.48 (1.38–1.58)	1.33 (1.24–1.42)	1.18 (1.09–1.28)
*P*					<0.001	<0.001	< 0.001
*P* for trend					<0.001	<0.001	< 0.001

*HR* hazard ratio, *CI* confidence interval, *BMI* body mass index *WC* waist circumference, *M* male, *F* female

^a^Incidence per 1000 person-years

^b^Model 1 was adjusted for age and sex

^c^Model 2 was adjusted for age, sex, smoking status, alcohol consumption, physical activity, income, estimated glomerular filtration rate, hypertension and diabetes mellitus

^d^Model 3 was adjusted for age, sex, smoking status, alcohol consumption, physical activity, income, estimated glomerular filtration rate, hypertension, diabetes mellitus and WC (or BMI)

### Risk of kidney cancer according to the coexistence of general and abdominal obesity

**Fig. 2:**
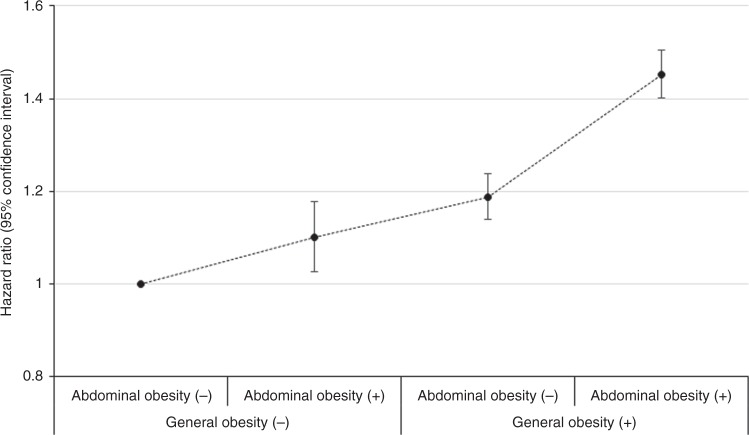
HR (95% CI) of kidney cancer according to the coexistence of general obesity and abdominal obesity (*P* < 0.001)

Figure 2 presents the HR (95% CI) of kidney cancer according to the combined presence of general obesity and abdominal obesity. Compared with the group without either type of obesity, the group with only abdominal obesity (HR 1.10, 95% CI: 1.03–1.18), the group with only general obesity (1.19, 1.14–1.24) and the group with both obesity types (1.45, 1.40–1.50) showed increased HRs of kidney cancer (*P* < 0.001).

### Risk of kidney cancer according to the presence of general and abdominal obesity in clinically relevant subgroups

In a subgroup analysis (Table 3), we observed the same trend of increasing risk of kidney cancer in individuals with general obesity or abdominal obesity compared to those without either of them. The association between general obesity and incident kidney cancer was more prominent in men (compared to that in women) and in the non-CKD group (compared to that in the CKD group) (*P* for interactions = 0.008 and 0.014, respectively). The association between abdominal obesity and incident kidney cancer was more prominent in the younger age group and in men, than it was in the older age group and in women, respectively (*P* for interactions < 0.001).

**Table 3:** HR (95% CI) of incident kidney cancer according to BMI and WC categories in clinically relevant subgroups^a^

	BMI (kg/m^2^)			WC (cm)		
	<25	≥25	*P* for interaction	<90 in men,<85 in women	≥90 in men, ≥85 in women	*P* for interaction
Age			0.172			<0.001
<65 years	1	1.33 (1.28–1.38)		1	1.36 (1.31–1.42)	
≥65 years	1	1.22 (1.15–1.29)		1	1.22 (1.15–1.29)	
Sex			0.008			<0.001
Men	1	1.38 (1.33–1.43)		1	1.38 (1.33–1.44)	
Women	1	1.28 (1.21–1.35)		1	1.24 (1.17–1.32)	
Hypertension			0.485			0.117
No	1	1.33 (1.28–1.39)		1	1.33 (1.27–1.40)	
Yes	1	1.28 (1.23–1.33)		1	1.30 (1.25–1.35)	
Diabetes mellitus			0.056			0.492
No	1	1.35 (1.30–1.39)		1	1.32 (1.28–1.37)	
Yes	1	1.20 (1.12–1.29)		1	1.31 (1.22–1.40)	
Dyslipidemia			0.627			0.789
No	1	1.32 (1.27–1.37)		1	1.31 (1.26–1.36)	
Yes	1	1.29 (1.22–1.36)		1	1.32 (1.25–1.40)	
Chronic kidney disease			0.014			0.171
No	1	1.35 (1.31–1.40)		1	1.33 (1.28–1.38)	
Yes	1	1.16 (1.07–1.26)		1	1.29 (1.19–1.41)	

*HR* hazard ratio, *CI* confidence interval, *BMI* body mass index, *WC* waist circumference

^a^HRs (95% CIs) were obtained using multivariable Cox proportional hazard regression analysis after adjusting for age, sex, smoking status, alcohol consumption, physical activity, income, estimated glomerular filtration rate, hypertension, diabetes mellitus

## Discussion

From this large longitudinal study, we found positive linear associations between BMI and WC, and the risk of incident kidney cancer. General obesity and abdominal obesity were associated with 1.32 times increased risk of incident kidney cancer and the combined presence of both type of obesity increased the risk by up to 1.45 times. Furthermore, the association between general obesity and abdominal obesity and risk of incident kidney cancer was more prominent in men than it was in women. Our results suggest that both general obesity and abdominal obesity are independently associated with incident kidney cancer and may be risk factors for kidney cancer in Korean adults. Our findings provide important information regarding the association between general obesity, abdominal obesity and kidney cancer in an East Asian population.

Obesity has been established as a risk factor for kidney cancer in Western countries. However, studies on the association between obesity and kidney cancer have produced inconsistent results and the issue remains to be elucidated in Asian populations. Prior Japanese population-based studies have reported either a U-shaped association between BMI and the risk of RCC,^17^ or a positive association between BMI and the risk of RCC-related death.^18^ In a Chinese population-based nested case control study, BMI was found to be associated with an elevated risk of RCC among men; the odds ratio corresponding to a 5 kg/m^2^ increase in BMI was 1.5 (95% CI: 1.1–2.0).^19^ A Korean prospective cohort study conducted in 2008 reported that the age-adjusted HRs for kidney cancer according to BMI groups were not significant (1.11, 95% CI: 0.93–1.31 for a group with BMI 25–29.9 kg/m^2^ and 1.38, 0.76–2.52 for a group with BMI ≥ 30 kg/m^2^, compared to a group with BMI 23–24.9 kg/m^2^) among men,^20^ contrary to our findings. Compared to previous studies, our study included a much larger sample size and considered various confounding variables, although the follow-up period was relatively short. We found positive associations between BMI and WC and kidney cancer, in the South Korean population; these associations were consistent among both men and women.

Although the precise mechanism of how obesity increases the risk of kidney cancer has not been identified, the following hypothesis may explain the link.^21,22^ Obesity is associated with hyperinsulinemia; insulin stimulates cell growth either directly through the insulin receptor, or through its ability to interact with the insulin-like growth factor-1 (IGF-1) receptor. Insulin resistance which is common in obesity and leads to an increase in the level of IGF-1. IGF-1 plays a role in carcinogenesis and leads to an increased risk of cancer. In addition, a Westernised lifestyle, such as consumption of high-fat and high-protein diet, and low physical activity may increase the incidence of RCC.^23–25^ Although specific dietary habits and nutrient intake were not assessed in our study, sedentary lifestyles including a currently smoking status, heavy alcohol drinking, and low physical activity were observed to be associated with a higher BMI (Table 1).

There is limited evidence linking abdominal obesity measured by WC and kidney cancer, and previous studies have reported conflicting findings. A report from the European Prospective Investigation into Cancer and Nutrition showed a positive association between WC and renal cancer; however, the number of renal cancer cases was small and moreover, the association was attenuated by adjustment for body weight.^26^ Two studies based on the Iowa Women’s Health Study reported an association between WC and risk of renal cancer, after adjustment only for age.^27,28^ A recent large cohort study of postmenopausal women reported stronger associations of kidney cancer with various obesity measures including WC than with BMI.^10^ Our findings suggest that WC is positively associated with kidney cancer even after a comprehensive adjustment for confounding variables including mutual adjustment for BMI. Positive association between abdominal obesity and kidney cancer risk can be supported by the findings that sole presence of abdominal obesity without general obesity was associated with 10% increased risk of kidney cancer shown in our study (Fig. 2). This finding also implies the possible impact of metabolic unhealthy status even in normal body weight on the incidence of kidney cancer. Measures of abdominal obesity have shown stronger associations with metabolic risk factors including insulin, than has BMI.^29^ Insulin resistance induced by abdominal adiposity results in hyperinsulinemia and suppresses hepatic production of hormonal binding proteins such as sex hormone-binding globulin (SHBG) and insulin-like growth factor binding protein (IGFBP).^30^ Consequently, excess adiposity increases circulating concentrations of total and bioavailable oestrogens, insulin, and bioavailable IGF-1, which promote carcinogenesis by enhancing proliferation of tissues and by inhibiting apoptosis.^21^ Visceral adipose tissue is associated with the increased release of various growth factors, chemokines, and cytokines that promote cancer development, including insulin-like growth factor and vascular endothelial growth.^31^ However, the association of kidney cancer risk was not observed to be stronger with WC than it was with BMI in our study, in contrast to the observations from the aforementioned previous study.^10^ Since the relatively short follow-up duration of our study seems not to be long enough to evaluate the effect of metabolic changes due to abdominal adiposity on kidney cancer incidence, further study is warranted to clarify this issue.

Interestingly, our study showed that the combined presence of general and abdominal obesity may have a synergistic effect in the development of kidney cancer. Coexistence of both conditions may have been associated with a higher incidence of obesity-related metabolic dysfunctions. Such dysfunctions may possibly be more strongly associated with an increase in risk of kidney cancer. Additionally, our study found more prominent associations between both general and abdominal obesity with kidney cancer in men than in women, in agreement with previous Western studies.

Some limitations should be addressed in interpreting our results. First, a retrospective design of our study cannot confirm a causal relationship between obesity, abdominal obesity and kidney cancer. Second, reverse causality cannot be ruled out, even though we considered washout period to account for the prodromal phase of kidney cancer. Third, the follow-up duration was relatively short to reflect metabolic change from obesity. Fourth, we could not assess the histologic subtype and severity of kidney cancer and information on dietary intake due to lack of data. Despite these limitations, the major strength of our study is the large sample size encompassing the entire South Korean population, allowing us to make comprehensive adjustments for confounding variables and to perform several types of subgroup analyses. We also focused on the association of abdominal obesity and combined presence of general and abdominal obesity contrast to previous studies. Furthermore, our results seem to provide additional important evidence regarding the association between obesity, abdominal obesity, and kidney cancer in an East Asian population.

In conclusion, BMI and WC were positively associated with the risk of kidney cancer, and general and abdominal obesity was associated with increased risk of kidney cancer in the entire South Korean population. Our results with providing important evidence for Asian population, suggest the need for better control of the growing epidemic of obesity and abdominal obesity, in order to prevent incident kidney cancer. Further study with longer duration of follow-up is needed to clarify the association.

## Supplementary information

HR (95% CI) of incident kidney cancer according to BMI and WC categories in men

HR (95% CI) of incident kidney cancer according to BMI and WC categories in women

## Data Availability

Data are available through the Korean National Health Insurance Sharing Service (NHISS). Researchers who wish to access the data can apply at (https://nhiss.nhis.or.kr/bd/ay/bdaya001iv.do).
