# Supplementary material for: Obesity, abdominal obesity and subsequent risk of kidney cancer: a cohort study of 23.3 million East Asians
Source: Br J Cancer. 2019 Jun 24;121(3):271–7. doi: 10.1038/s41416-019-0500-z (PMC6738324; doi:10.1038/s41416-019-0500-z)
Supplement: Supplementary file 1 — HR (95% CI) of incident kidney cancer according to BMI and WC categories in men [file 41416_2019_500_MOESM1_ESM.docx]

**Supplemental Table 1.** HR (95% CI) of incident kidney cancer according to BMI and WC categories in men

|  | N | Event | Person-years | Incidence rate^a^ | HR (95% CI) | | |
| --- | --- | --- | --- | --- | --- | --- | --- |
|  |  |  |  |  | Model 1^b^ | Model 2^c^ | Model 3^d^ |
| BMI (per 1 kg/m^2^) |  |  |  |  | 1.088 (1.082–1.094) | 1.070 (1.062–1.079) | 1.070 (1.063–1.076) |
| BMI (kg/m^2^) |  |  |  |  |  |  |  |
| <25.0 | 7,428,797 | 6875 | 40,279,494 | 0.17 | 1 (ref.) | 1 (ref.) | 1 (ref.) |
| ≥25.0 | 4,400,096 | 5662 | 23,826,775 | 0.24 | 1.51 (1.45-1.56) | 1.41 (1.34-1.49) | 1.38 (1.33-1.43) |
| P |  |  |  |  | <0.001 | <0.001 | <0.001 |
| <18.5 | 8,423,818 | 3289 | 44,956,926 | 0.07 | 0.78 (0.68–0.90) | 0.59 (0.49–0.72) | 0.83 (0.72–0.96) |
| 18.5–22.9 | 3,060,335 | 2210 | 16,458,492 | 0.13 | 1 (ref.) | 1 (ref.) | 1 (ref.) |
| 23.0–24.9 | 282,053 | 204 | 1,466,908 | 0.14 | 1.33 (1.27–1.39) | 1.27 (1.18–1.36) | 1.27 (1.21–1.33) |
| 25.0–29.9 | 3,987,644 | 3235 | 21,582,153 | 0.15 | 1.66 (1.58–1.73) | 1.45 (1.36–1.55) | 1.51 (1.44–1.58) |
| ≥30.0 | 3,159,100 | 3436 | 17,230,432 | 0.20 | 2.30 (2.10–2.51) | 2.01 (1.80–2.25) | 1.93 (1.77–2.11) |
| P |  |  |  |  | <0.001 | <0.001 | <0.001 |
| P for trend |  |  |  |  | <0.001 | <0.001 | <0.001 |
| WC (per 5 cm) |  |  |  |  | 1.188 (1.175–1.201) | 1.137 (1.120–1.155) | 1.151 (1.138–1.164) |
| WC (cm) |  |  |  |  |  |  |  |
| M <90.0, F <85.0 | 3,949,283 | 5064 | 21,435,957 | 0.24 | 1 (ref.) | 1 (ref.) | 1 (ref.) |
| M ≥90.0, F ≥85.0 | 450,813 | 598 | 2,390,818 | 0.25 | 1.53 (1.47-1.59) | 1.38 (1.30-1.47) | 1.38 (1.33-1.44) |
| P |  |  |  |  | <0.001 | <0.001 | <0.001 |
| <80.0 | 663,806 | 104 | 3,463,512 | 0.03 | 0.61 (0.58–0.65) | 0.67 (0.62–0.73) | 0.67 (0.63–0.70) |
| 80.0–84.9 | 5,281,937 | 1802 | 28,114,539 | 0.06 | 0.84 (0.80–0.88) | 0.96 (0.89–1.04) | 0.86 (0.82–0.91) |
| 85.0–89.9 | 2,478,075 | 1383 | 13,378,876 | 0.10 | 1 (ref.) | 1 (ref.) | 1 (ref.) |
| 90.0–94.9 | 2,646,867 | 1846 | 14,279,813 | 0.13 | 1.13 (1.08–1.20) | 1.09 (1.004–1.20) | 1.10 (1.04–1.16) |
| 95.0–99.9 | 413,468 | 364 | 2,178,679 | 0.17 | 1.34 (1.26–1.44) | 1.24 (1.12–1.38) | 1.26 (1.18–1.35) |
| ≥100.0 | 9,190,551 | 8331 | 49,915,036 | 0.17 | 1.56 (1.44–1.70) | 1.39 (1.24–1.57) | 1.41 (1.29–1.53) |
| P |  |  |  |  | <0.001 | <0.001 | <0.001 |
| P for trend |  |  |  |  | <0.001 | <0.001 | <0.001 |

Abbreviations: HR, hazard ratio; CI, confidence interval; BMI, body mass index; WC, waist circumference; M, male; F, female.

^a^ Incidence per 1000 person-years

^b^ Model 1 was adjusted for age and sex.

^c^ Model 2 was adjusted for age, sex, smoking status, alcohol consumption, physical activity, income, estimated glomerular filtration rate, hypertension, and diabetes mellitus.

^d^ Model 3 was adjusted for age, sex, smoking status, alcohol consumption, physical activity, income, estimated glomerular filtration rate, hypertension, diabetes mellitus, and WC (or BMI).
