# Supplementary material for: Obesity, abdominal obesity and subsequent risk of kidney cancer: a cohort study of 23.3 million East Asians
Source: Br J Cancer. 2019 Jun 24;121(3):271–7. doi: 10.1038/s41416-019-0500-z (PMC6738324; doi:10.1038/s41416-019-0500-z)
Supplement: Supplementary file 2 — HR (95% CI) of incident kidney cancer according to BMI and WC categories in women [file 41416_2019_500_MOESM2_ESM.docx]

**Supplemental Table 2.** HR (95% CI) of incident kidney cancer according to BMI and WC categories in women

|  | N | Event | Person-years | Incidence rate^a^ | HR (95% CI) | | |
| --- | --- | --- | --- | --- | --- | --- | --- |
|  |  |  |  |  | Model 1^b^ | Model 2^c^ | Model 3^d^ |
| BMI (per 1 kg/m^2^) |  |  |  |  | 1.054 (1.046–1.062) | 1.037 (1.027–1.046) | 1.046 (1.034–1.058) |
| BMI (kg/m^2^) |  |  |  |  |  |  |  |
| <25.0 | 2,638,342 | 4206 | 14,191,233 | 0.30 | 1 (ref.) | 1 (ref.) | 1 (ref.) |
| ≥25.0 | 9,420,636 | 3783 | 50,335,870 | 0.08 | 1.28 (1.21-1.35) | 1.12 (1.07-1.17) | 1.11 (1.04-1.19) |
| P |  |  |  |  | <0.001 | <0.001 | <0.001 |
| <18.5 | 2,063,517 | 1716 | 11,079,549 | 0.16 | 0.61 (0.50–0.74) | 0.92 (0.79–1.06) | 0.65 (0.53–0.79) |
| 18.5–22.9 | 3,457,031 | 2146 | 18,669,226 | 0.12 | 1 (ref.) | 1 (ref.) | 1 (ref.) |
| 23.0–24.9 | 3,082,755 | 2926 | 16,820,556 | 0.17 | 1.22 (1.13–1.31) | 1.13 (1.07–1.19) | 1.12 (1.04–1.21) |
| 25.0–29.9 | 2,650,765 | 3259 | 14,425,253 | 0.23 | 1.32 (1.23–1.41) | 1.21 (1.14–1.29) | 1.18 (1.08–1.28) |
| ≥30.0 | 1,606,385 | 2363 | 8,689,994 | 0.27 | 1.67 (1.48–1.87) | 1.35 (1.21–1.52) | 1.44 (1.24–1.67) |
| P |  |  |  |  | <0.001 | <0.001 | <0.001 |
| P for trend |  |  |  |  | <0.001 | <0.001 | <0.001 |
| WC (per 5 cm) |  |  |  |  | 1.103 (1.086–1.121) | 1.119 (1.100–1.138) | 1.061 (1.036–1.085) |
| WC (cm) |  |  |  |  |  |  |  |
| <85.0 | 680,134 | 1185 | 3,646,615 | 0.33 | 1 (ref.) | 1 (ref.) | 1 (ref.) |
| ≥85.0 | 351,823 | 658 | 1,854,624 | 0.36 | 1.24 (1.17-1.32) | 1.16 (1.10-1.21) | 1.05 (0.97-1.13) |
| P |  |  |  |  | <0.001 | <0.001 | <0.001 |
| <75.0 | 5,233,698 | 1384 | 27,787,736 | 0.05 | 0.72 (0.67–0.79) | 0.75 (0.70–0.80) | 0.83 (0.75–0.91) |
| 75.0–79.9 | 2,289,330 | 1200 | 12,303,584 | 0.10 | 1.00 (0.92–1.08) | 0.91 (0.86–0.96) | 1.05 (0.96–1.14) |
| 80.0–84.9 | 1,897,608 | 1199 | 10,244,550 | 0.12 | 1 (ref.) | 1 (ref.) | 1 (ref.) |
| 85.0–89.9 | 1,114,102 | 838 | 6,011,549 | 0.14 | 1.06 (0.97–1.16) | 1.06 (1.00–1.12) | 1.02 (0.94–1.12) |
| 90.0–94.9 | 576,824 | 515 | 3,098,689 | 0.17 | 1.16 (1.04–1.29) | 1.18 (1.10–1.27) | 1.08 (0.96–1.20) |
| ≥95.0 | 372,591 | 363 | 1,969,312 | 0.18 | 1.24 (1.10–1.40) | 1.27 (1.15–1.40) | 1.07 (0.93–1.23) |
| P |  |  |  |  | <0.001 | <0.001 | <0.001 |
| P for trend |  |  |  |  | <0.001 | <0.001 | <0.001 |

Abbreviations: HR, hazard ratio; CI, confidence interval; BMI, body mass index; WC, waist circumference; M, male; F, female.

^a^ Incidence per 1000 person-years

^b^ Model 1 was adjusted for age and sex.

^c^ Model 2 was adjusted for age, sex, smoking status, alcohol consumption, physical activity, income, estimated glomerular filtration rate, hypertension, and diabetes mellitus.

^d^ Model 3 was adjusted for age, sex, smoking status, alcohol consumption, physical activity, income, estimated glomerular filtration rate, hypertension, diabetes mellitus, and WC (or BMI).
